# Proceedings of the Society of Dental Surgeons of the State of New York

**Published:** 1850-01

**Authors:** 


From the New York Dental Recorder.

### PROCEEDINGS OF THE SOCIETY OF DENTAL SURGEONS

### *of the State of New York.*

A regular meeting of this society was held at its own rooms in Broadway, on the evening of Tuesday, Dec. 4th, the President, Dr. Lovejoy in the chair.

After the reading of the minutes of the previous meeting, the committee, appointed to confer with Mr. James Alcock, reported that he was ready to fulfil his promise, to present an instrument case to the Society, and only waited for the committee on clinical practice to suggest the pattern and style which would be most convenient for the Dental Infirmary. The report was accepted, aud the thanks of the Society conferred upon Mr. Alcock.

The report of the Treasurer was then laid before the Society, and the names of those who had not paid their annual dues for 1848, 1849, ordered to be read. The Secretary was ordered to notify the delinquent members, in conformity with the by-laws of the Society.

Essays and addresses being next in order, Mr. L. Covell, was requested to read an address, which he had voluntarily prepared for this occasion. The address of Mr. C. on the subject of Mutual Improvement among Dentists, was listened to with pleasing attention, after which, the the thanks of the Society were voted to him.

On motion of F. H. Clark, the report of the committee "on the expediency of granting diplomas or certificates of membership," made at the last annual meeting, was called up and discussed by the Society, after which C. C. Allen moved to commit it to a special committee, with instructions to report resolutions in favor of granting diplomas or certificates of membership to its members. This motion was adopted, and Allen, Lord, and Chase appointed.

On motion of Mr. L. Covell, a committee was appointed to revise the rules of order.

The Society then adjourned to meet on the evening of December 17.

At the adjourned meeting, Dec. 17, after the preliminaries of organization, reports of committees being in order, Dr. Harvey Burdell, from the committee on "the best mode of taking impressions," read a lengthy and minute report, which was accepted, and the committee discharged.

The special committee on diplomas and certificates of membership, reported the following resolutions :

*Resolved*, That any member of this Society, who shall present himself before a committee, to be appointed by the Society, for the purpose of examining members and candidates for membership, and who, after a thorough examination in the theory and practice of Dental Surgery, shall be pronounced, before the Society, by the majority of said committee, well qualified to assume the duties and responsibilities of a Dental Surgeon, shall, a majority of the members present concurring, on his paying into the treasury the sum of ten dollars, be entitled to a diploma, recommending him as one worthy the confidence and patronage of the public.

*Resolved*, That each member of the Society shall be entitled, annually, to a certificate of membership, printed from a handsome copper-plate engraving, which shall be his receipt for annual, and all other dues to this Society.

*Resolved*, That art. 2d, sec. 3d of the by-laws of this Society be amended by adding the words "*Examining, or*" immediately before the words "Executive Committee," and that sec. 4th be amended, by adding the words, *and practice*, immediately after the word "theory."

It was then moved that the resolutions be adopted. Mr. F. H. Clark asked for a division, and the question being taken on each resolution separately, they were all adopted by the Society.

The committee appointed to revise the Rules of Order, reported through Mr. Covell, and after some discussion, the revised rules were adopted, and ordered to be printed, with the constitution and by-laws.

A committee was then appointed, consisting of Burdell, Clark and Rowell, to which were added the President and Secretary, to submit to the Society drafts of diplomas, and certificates of membership.

On motion, the Executive Committee were ordered to have printed 250 copies of the Constitution, By-laws and Rules of Order for the use of the members.

Adjourned until the evening of January 14, 1850.

